# Intervention in Older Urban-Dwelling Veterans With Dysmobility: Protocol for a Pilot Feasibility Clinical Trial

**DOI:** 10.2196/39192

**Published:** 2022-07-13

**Authors:** Ronna N Robbins, Monica C Serra, Lisa S Kilpela, Elizabeth A Parker, Rozmin Jiwani, Odessa Addison

**Affiliations:** 1 San Antonio Geriatric, Research, Education, and Clinic Center South Texas Veterans Health Care System San Antonio, TX United States; 2 Division of Geriatrics, Gerontology and Palliative Medicine and Barshop Institute for Longevity & Aging Studies, Department of Medicine University of Texas Health Science Center San Antonio, TX United States; 3 Department of Psychiatry & Behavioral Sciences and Barshop Institute for Longevity & Aging Studies University of Texas Health Science Center San Antonio, TX United States; 4 Baltimore Geriatric Research, Education, and Clinical Center VA Maryland Health Care System Baltimore, MD United States; 5 Department of Physical Therapy and Rehabilitation Science University of Maryland School of Medicine Baltimore, MD United States; 6 School of Nursing University of Texas Health Science Center San Antonio, TX United States

**Keywords:** peer-led, veterans, dysmobility, lifestyle modification programs

## Abstract

**Background:**

The majority of older veterans do not meet the minimum healthy diet or physical activity recommendations despite known benefits. Identifying ways to increase adherence to programs that improve dietary quality and physical activity may reduce the risk of disability in older veterans. Peer-based interventions may be one method for facilitating lasting behavior change because peers often share a common culture and knowledge regarding problems their community experiences.

**Objective:**

This study aims to develop, pilot, and evaluate a theory-driven, 12-week, peer-led nutrition and exercise intervention that targets older veterans with dysmobility and assess its feasibility in 2 diverse urban areas with underrepresented populations.

**Methods:**

Community-dwelling veterans aged >65 years with self-reported dysmobility (defined as difficulty in at least 1 of the following: walking quickly across a street, walking a mile, ascending a flight of stairs, rising from a chair without the use of arms, or a fear of falling) from 2 Department of Veterans Affairs Geriatric Research, Education, and Clinic Centers (Baltimore, Maryland, and San Antonio, Texas) will be eligible to participate. First, this study will use validated mixed methods via web-based surveys (n=50 per site) to assess potential physical, social or environmental, and behavioral or lifestyle barriers that affect physical activity and dietary quality (phase 1). Next, we will use knowledge gained from these assessments and feedback from a focus group (n=10 per site) to adapt established Department of Veterans Affairs diet and exercise program materials to develop peer-led intervention materials and train peer leaders (n=3 per site). Finally, we will determine the feasibility and acceptability of the intervention to assess reach (recruitment and retention), adoption (satisfaction, perceived utility, attendance, and engagement), and implementation (fidelity of intervention), as well as the estimated magnitude and potential impact on selected outcomes (ie, diet quality and mobility) in 20 older veterans with dysmobility (n=10 per site).

**Results:**

The study was funded on January 1, 2022, with a projected data collection period of June 1, 2022, to December 31, 2023.

**Conclusions:**

This study offers an innovative approach to identifying strategies that increase long-term adherence to lifestyle modification programs that improve dietary quality and physical activity in older veterans with dysmobility.

**Trial Registration:**

ClinicalTrials.gov NCT04994938; https://clinicaltrials.gov/ct2/show/NCT04994938

**International Registered Report Identifier (IRRID):**

PRR1-10.2196/39192

## Introduction

### Background

US veterans are a multifaceted population with unique health challenges. The veteran population is disproportionately older men (>50% are aged >65 years) [1] who have a higher prevalence of obesity [2], multimorbidity [3], and self-reported disability or dysmobility [4] and have suboptimal dietary quality and perform less physical activity than nonveterans [5]. Decades of research has demonstrated that exercise is an effective intervention to improve mobility and overall health [6]. Despite these known benefits, the majority of older veterans do not meet the minimum physical activity recommendations for either aerobic or resistance exercises [7]. The ability to safely maintain mobility with age is critical, as immobility is the leading cause of long-term care admissions and increases fall risk, health care use, and expenditure [8]. Older adults with mobility limitations are also more likely to have poor diet quality [9]. Furthermore, as veterans are at increased risk of obesity and dysmobility, they are also more likely to have lower diet quality and deviate further from dietary guidelines than nonveterans [10]. Poor diet quality is primarily due to high consumption of empty calories from added sugar and solid fats and lower intake of fruits, vegetables, whole grains, and dairy [10]. Many well-established factors contribute to poor nutrition in aging, including physiological, social, emotional, and environmental changes [11-13]. Among older veterans with mobility limitations, these factors are further compounded by accessibility limitations and the inability to complete instrumental activities of daily living such as shopping or cooking [14].

Chronic disease and frailty are likely to develop with age, and these outcomes are associated with an increased risk of poor quality of life [15]. A recent systematic review reported strong and consistent observational evidence for a link between *healthier* diets and a lower risk of decline in physical performance [16]. Furthermore, exercise interventions in veterans have consistently been shown to improve physical performance [17]. Veteran participation in the Department of Veterans Affairs (VA) MOVE! Weight Management Program, the largest weight management program in the United States, is associated with successful short-term weight loss and greater weight loss as participation engagement increases [18]. Participation in Gerofit, a national VA exercise and health promotion program targeting older veterans with dysmobility, is associated with improved mobility [17]. Even professionally led, evidence-based lifestyle intervention programs that have a positive impact on weight management and mobility have low long-term adherence rates [19]. Therefore, identifying strategies that increase long-term adherence to programs that improve the dietary intake quality and physical activity of older veterans may reduce the risk of disability by maintaining mobility and preserving cardiovascular health with advancing age.

### Objective

Peer-based interventions may be one method of facilitating lasting health behavior change, because peers often share a common culture, language, and knowledge about the problems their community experiences [20]. Group-based, peer-led lifestyle interventions are particularly well suited for older veterans, as social interaction is a powerful motivator [21]. Although previous peer-led diet and exercise interventions have been successfully implemented [22], there are numerous limitations in their design that make them suboptimal for older veterans with dysmobility. The majority of these studies targeted only diet or exercise and did not emphasize the achievement of national dietary guidelines [22]. In addition, few studies have focused directly on the unique needs of older veterans who often live in underserved areas without access to professional resources [22]. This is especially important to address, as underrepresented minority populations are projected to constitute >35% of the veteran population by 2040 [23]. These gaps in the literature provide opportunities for improvement in peer-led interventions. Our central hypothesis is that we can develop, pilot, and evaluate a 12-week, peer-led lifestyle intervention targeting older veterans in underserved minority populations with dysmobility.

## Methods

### Study Design and Overview

**Figure 1:**
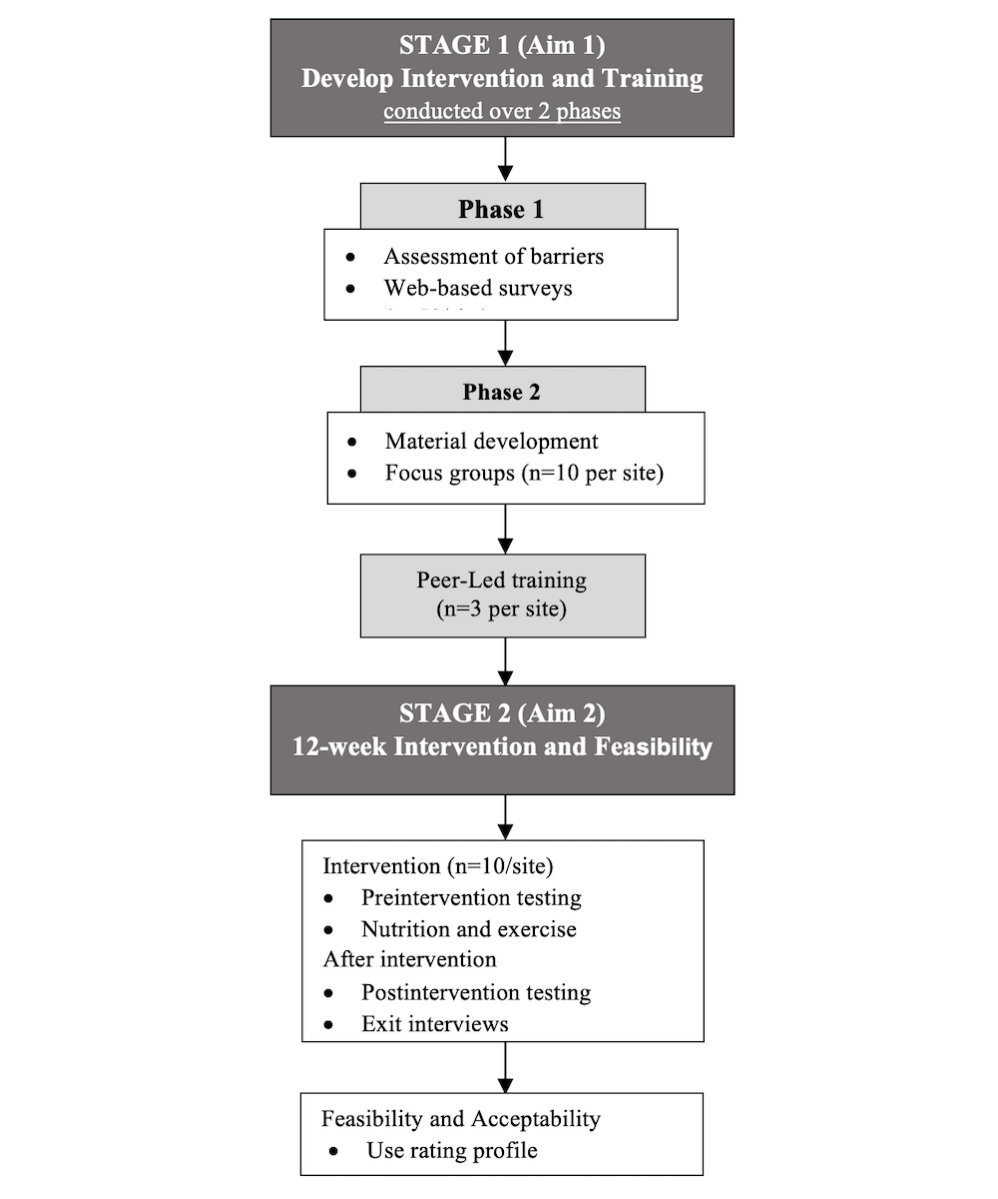
Study design.

This is a multisite feasibility clinical trial pilot study. Figure 1 shows the study design, and Table 1 shows the study timeline. The study protocol follows the SPIRIT (Standard Protocol Items: Recommendations for Interventional Trials) guidelines [24]. Any deviations from the protocol, breaches of confidentiality, and reportable adverse events will be reported to the respective institutional review boards (IRBs) and data safety monitoring boards according to local policies. In addition, the data safety monitoring boards will review study-related materials at least annually and review the collected data to ensure data integrity, security, and control for quality assurance. The study is registered at *ClinicalTrials.gov* (NCT04994938). The proposed study will develop a peer-led nutrition and exercise intervention (aim 1) and will pilot and evaluate its feasibility, acceptance, and impact (aim 2) in 2 diverse urban areas with high underrepresented populations of older veterans (Baltimore, Maryland, and San Antonio, Texas). Each site will conduct the study in parallel, and the study protocol has been approved by the local IRB and the VA Research and Development Committees. As seen in Figure 1, the study is designed to be conducted over 2 stages. Stage 1 will develop a theory-driven, peer-led nutrition and exercise intervention tailored for older veterans with dysmobility (aim 1), whereas stage 2 will evaluate the feasibility, acceptability, and impact of a peer-led pilot intervention (aim 2).

**Table 1:** Study timeline.

				Yearly quarters															
				1 (n=10 per site)		2 (n=20 per site)		3 (n=20 per site)		4 (n=10 per site)			5 (n=3 per site)		6 (n=5 per site)		7 (n=5 per site)		8
**Stage 1 (aim 1)**																			
	Phase 1: web-based survey		✓		✓		✓												
	**Phase 2**																		
		Material development	✓		✓		✓												
		Focus groups									✓								
		Peer training										✓							
**Stage 2 (aim 2)**																			
	Pretesting													✓					
	Intervention													✓		✓			
	Posttesting															✓			
	Feasibility																	✓	

### Study Design Stage 1 (Aim 1)

#### Overview

In stage 1, peer-led nutrition and exercise intervention will be achieved through a mixed methods approach that uses a concurrent nested design [25] over the following two phases: (1) phase 1 (Assessment of Barriers): using validated quantitative and qualitative assessments implemented via web-based surveys, potential physical, social or environmental, and behavioral or lifestyle barriers and facilitators that affect activity and nutrition will be assessed and (2) phase 2 (Program Adaptation and Peer Leader Training): using a task-shifting approach [26], the current VA MOVE! and Gerofit programs will be adapted based on knowledge gained in phase 1 and through focus groups to develop peer-led training materials and train peer leaders.

#### Phase 1: Assessment of Barriers and Facilitators

##### Participants and Inclusion and Exclusion Criteria

Given the web-based nature of this aim, the inclusion and exclusion criteria are minimal, and anonymous web-based surveys are exempt under local IRB guidelines. Community-dwelling veterans (male and female) aged >65 years (n=50 per site) with self-reported dysmobility (defined as difficulty in at least 1 of the following activities: walking quickly across a street, walking a mile, ascending a flight of stairs, rising from a chair without the use of arms, or a fear of falling) and the ability to take the survey in English will be recruited to complete web-based surveys.

##### Recruitment and Screening

Participants will be recruited from the Baltimore (Baltimore, Maryland) and Audie Murphy (San Antonio, Texas) Veterans Administration Medical Centers (VAMCs), local Geriatric Research Education Clinics Centers’ registries and contacts, local veteran groups, posts in web-based forums, media advertisements, and finally through word of mouth. Those responding to recruitment efforts will be telephonically screened and asked a series of questions to determine eligibility. Individuals who meet the eligibility criteria will be invited to complete the web-based surveys.

##### Web-Based Surveys, Questionnaires, and Assessments

Eligible participants will provide demographic information and will self-report their medical history, current medications, and height and weight. Participants will also complete 25 to 30 minutes of validated questionnaires (Table 2) administered via the VA-approved Federal Risk and Authorization Management Program version of Qualtrics to assess potential physical (medical morbidities and physical mobility), social or environmental (food insecurity and access to exercise or recreation), and behavioral or lifestyle (physical activity, sleep disturbances, and use of television, internet, and alcohol) barriers and facilitators that may affect physical activity and dietary quality in older adults with dysmobility. In addition, participants will be provided with log-in information and asked to complete a 24-hour dietary recall [27] for the previous day using the Automated Self-Administered 24-Hour Dietary Assessment Tool [28]. Food records will be used to calculate the Healthy Eating Index score, a measure of dietary quality used to assess adherence to national dietary guidelines [29].

**Table 2:** Aim 1: quantitative and qualitative measures.

Assessment measures				Description
**Primary correlates**				
	Physical activity (Physical Activity Scale for the Elderly)		A 11-item self-report of physical activity: leisure time and household activities and optional work and volunteer activities. Good reliability, validity; brief to reduce participant burden [30].	
	Dietary intake quality (Automated Self-Administered 24-hour Dietary Assessment)		Validated web-based tool developed by the National Cancer Institute that enables multiple, automatically coded, and self-administered 24-hour diet recalls. The recall will be used to calculate the HEI^a^. HEI scores range from 0 to 100, with higher scores indicating better adherence to the Dietary Guidelines for Americans [28,31].	
**Quantitative assessments**				
	BMI		Self-reported height and weight (owing to survey nature of study); calculated as weight (kg) and height squared (m^2^).	
	Demographics and history		Self-reported annual household income, education, and marital status and medical history or comorbidity, polypharmacy (ie, number of current medications), and surgeries.	
	**Eating behaviors**			
		Short Healthy Eating Index	Measure of diet quality used to assess how well dietary patterns align with key recommendations of the Dietary Guidelines for Americans [32].	
		A 3-factor eating questionnaire	A 21-item questionnaire that measures 3 domains of eating behavior: cognitive restraint, uncontrolled eating, and emotional eating [33].	
		Department of Veterans Affairs binge eating screener	A single question, validated in veterans, that assesses the frequency of binge eating [33].	
		Barriers to diet and exercise	A total of 12 questions used in the Life Trial that assessed potential barriers and the extent of those barriers that make it difficult to change eating and exercise habits [34].	
		Short Food Security Scale	A 6-item survey, developed by the USDA^b^ that identifies food-insecure households and households with very low food security [35].	
	**Health and lifestyle behaviors**			
		Short Form Survey-12	A 12-item validated Quality of Life Questionnaire that measures 8 health domains: physical function, pain, role limitations owing to physical health problems, personal or emotional problems, emotional well-being, social functioning, energy and fatigue, and general health perceptions [36].	
		Alcohol Use Disorders Identification Test-3	A 10-item screening tool to assess alcohol consumption, drinking behaviors, and alcohol-related problems [37].	
		Television and internet use	Will be assessed through 2 multiple-choice questions: (1) On average, how long do you spend using a computer, tablet, or phone to be on the internet (eg, reading news, playing games, or watching shows) per day? and (2) How long do you spend watching television or movies per day? Response options range from none to >12 hours.	
		Sleep disturbance (Insomnia Severity Index-7)	A 7-item questionnaire to assess the nature, severity, and impact of insomnia and monitor treatment response in adults [38].	
		Depression (Center for Epidemiologic Studies Depression Scale)	A 20-item screening test for depression and depressive disorder. The CES-D^c^ measures symptoms defined by the American Psychiatric Association Diagnostic and Statistical Manual for a major depressive episode [39].	
**Qualitative assessments**				
	Eating and physical activity [40]		What were the eating and physical activity habits you had before, during, and after your military service? While thinking of all these times in your life before, during, and after your military service, what eating and physical activity habits stand out to you? What do you think would help you to be more physically active? Some veterans say that they eat when they experience stress or think of things that are hard to deal with; has this ever happened to you? Can you tell me about it? What helps you the most to eat healthy and exercise? What is the biggest barrier to eating healthy and exercising?	
	Cultural or contextual factors		What are the local norms around the perceptions of food (or meals) and physical activity in your community? How are *favorite* foods prepared and served? What are local meal patterns and food preferences? What are local perceptions of preferred body size? What value is placed on fatness?	

^a^HEI: Healthy Eating Index.

^b^USDA: United States Department of Agriculture.

^c^CES-D: Center for Epidemiologic Studies Depression Scale.

At the end of the web-based quantitative assessment, participants will also be asked to schedule an interview with a research team member (MSC, LSK, or OA) to complete a qualitative assessment. The qualitative interview will occur via telephone or web-based assessment and will provide an opportunity to delve deeper into individual barriers and facilitators of healthy diet and exercise habits. The qualitative assessment questions are presented in Table 2. A concurrent nested mixed methods design will be used to conduct both quantitative and qualitative assessments in the same phase. This embedded design will allow the gathering of quantitative data on the constructs of interest, and then, using open-ended qualitative questions, more in-depth information about the data will be gathered. This embedded design uses qualitative data in a supportive role to better explain the relationships gathered from quantitative data [25].

##### Phase 1 Outcomes and Analysis

We will identify quantitative factors in Table 2 that correlate with low physical activity (assessed via the Physical Activity Scale for the Elderly) [30] and poor diet quality (Healthy Eating Index assessed via a validated questionnaire [32] and calculated from the Automated Self-Administered 24-Hour Dietary Assessment Tool) [28] using multiple linear regression. Qualitative analysis will be used to identify themes (see *Phase 2: Program Adaptation and Peer Leader Training* for further details). Integration of this aim will involve connecting quantitative results with qualitative findings.

#### Phase 2: Program Adaptation and Peer Leader Training

##### Overview

On the basis of the knowledge gained from phase 1, the VA MOVE! and Gerofit programs, which are currently running successfully at the Baltimore and San Antonio sites, will be adapted to develop peer leader training materials that target older veterans. Using a task-shifting approach [41], we will modify available handouts and participant resources from the MOVE! and Gerofit. Once the materials are drafted, focus groups will be conducted to provide feedback on the developed program.

##### Focus Group Inclusion and Exclusion Criteria, Recruitment, and Screening

A mixed group of veterans (male and female; aged >65 years) who are actively engaged in lifestyle change programs such as MOVE! or Gerofit (n=10 per site) and meet the inclusion criteria in phase 1 will be recruited to participate in the focus groups.

##### Focus Groups

Before completing any data collection, all focus group participants will provide written informed consent and complete the Health Insurance Portability and Accountability Act (HIPAA) authorization form. Focus groups will be led by the investigators (in-person when on-site and web-based when off-site) and will gather feedback using evidence-based materials on the content and presentation of materials, rating of acceptability, and relevance. We will ask for their input on the positives, negatives, and what was left out. In addition, we will ask them to share their favorite and least favorite parts (defined as what they liked, thought was most relevant, or thought would be most helpful and vice versa for least favorite options). Finally, we gather information on logistical considerations (eg, group vs one-on-one, in-person vs virtual, apps or trackers, frequency of meetings, and duration).

For cultural tailoring, we will consider family and community dynamics, access to resources (food and activity), materials goodness of fit (eg, written words sit better with some, whereas graphics with others or individualistic vs collectivistic cultural approaches to interventions), ways to reach the communities of interest, and language.

##### Outcomes and Analysis

Focus group interviews will be recorded and transcribed, and a summary template will be developed to gather key points from the interview guide. The transcripts will be coded to identify themes (eg, reported experiences) using qualitative analysis software (Atlas.ti; Scientific Software Development GmbH), review patterns in core themes, determine the degree of overlap, and develop a network diagram of interrelationships between themes. The themes will be used to describe the barriers and facilitators of diet quality, energy balance, and physical activity (Table 2) that may influence treatment.

##### Peer Leader Inclusion and Exclusion Criteria, Recruitment, and Training

Once materials are finalized according to focus group feedback, peer leaders (n=3 per site) will be recruited at each site from the focus groups to undergo training. Peer leaders will be selected based on (1) prior participation in a VA-directed lifestyle program for at least six months, (2) demonstration of an understanding of the importance of diet and exercise determined by a successful diet change, and (3) expression of a desire for further training in the peer leader role when approached [42]. In addition, because peer leaders will serve as aspirational behavioral role models [43], they will be selected from veterans who have been successful in making and maintaining positive changes to their diet and exercise habits and are familiar with community networks and group facilitation. Furthermore, studies suggest that peer leaders of older adults should be optimistic, inclusive, and compassionate [44]; therefore, we will use these qualities along with the inclusion criteria listed earlier to select and train peer leaders using need-supportive motivation strategies [43].

##### Peer Leader Training

Once chosen, peer leaders will provide written informed consent, complete a HIPAA authorization form, and attend a full-day virtual training workshop with peer leaders and research team members from both sites. Training will be web-based and informal, with emphasis on the provision of social support to encourage positive behavior change. A research team member with expertise in developing peer interventions will oversee the development of peer training. Peer leaders will learn the importance of physical activity and healthy eating. They will learn about the social determinants of health and discuss solutions to overcome potential physical, social or environmental, and behavioral or lifestyle barriers described among older veterans in their communities (Table 2). They will also be provided with key safety information necessary for working with older veterans with comorbid conditions (eg, diabetes) and indicators that an exercise intervention should be discontinued (eg, signs of low blood sugar). In addition, peer leaders will learn key communication skills to convey information to their peers as advocates of change. Training will consist of leading mock sessions and receiving supervision from a research team member. Mock sessions will be recorded, and the sessions will be rated against an adherence checklist.

### Study Design Stage 2 (Aim 2)

Stage 2 will determine the feasibility and acceptability and the estimated magnitude of the potential impact on selected primary and secondary outcomes of the peer-led diet and exercise pilot intervention in older veterans with dysmobility.

#### Participants and Inclusion and Exclusion Criteria

Community-dwelling veterans (male and female) aged >65 years (n=10 per site) who self-identify as having dysmobility (same inclusion as phase 1) will be included. Exclusion criteria include (1) high cardiovascular risk (poorly controlled hypertension >160/100 mm Hg, class IV chronic heart failure, symptomatic angina at rest, or syncope in the past year without known resolution of cause); (2) use of home oxygen; (3) contraindications to an exercise intervention; (4) dementia (on medical record review or a mini-mental status exam score <25); (5) currently regularly exercising or participating in a diet or weight loss intervention; and (6) behavior that prevents group interaction.

#### Recruitment and Screening

Participants will be recruited using the same strategies as in phase 1. Individuals responding to recruitment efforts will initially be telephonically screened to assess their eligibility and potential interest in enrollment in the study. Individuals who pass the screening will be invited to the respective facility to sign informed consent; complete a HIPAA authorization form; provide demographics, brief medical history, and medications; and then undergo a physical examination. A Montreal Cognitive Assessment examination will be performed as part of the physical examination to screen for dementia. Participants with a Montreal Cognitive Assessment score <25 will be excluded. Participants who remain eligible will be scheduled for baseline assessments.

#### Baseline Assessments Measures and Subjective Assessments

A baseline assessment battery will occur before the beginning of and after the 12-week, peer-led nutrition and exercise pilot intervention. All baseline assessments and tests will be conducted at the respective facilities (Baltimore and Audie Murphy VAMCs) over 1 to 2 visits during a 1-week period and will be collected using standardized protocols and trained research team members. These assessments are described in detail in Textbox 1 and include the following domains: body composition, caloric balance, and physical function. Assistive devices will be used during the assessments of physical function as needed, with the same device used at baseline and follow-up assessments. In addition, participants will complete web-based subjective assessments (eg, surveys and questionnaires) administered via VA-approved Federal Risk and Authorization Management Program version of Qualtrics to assess potential physical, social or environmental, and behavioral or lifestyle barriers and facilitators that may affect physical activity and dietary intake quality in older adults with dysmobility. The same uestionnnaires outlined in stage 1 will be used in stage 2 (Table 2).

**Aim 2: baseline and postintervention assessment measures. Assistive devices will be used as needed, with the same device used at baseline and the follow-up assessment.**

**Assessment measure and description**

- BMI: weight (kg) will be determined at baseline and weekly during the 12-week intervention with participants dressed in light clothing without shoes. Standing height and weight will be measured using a calibrated digital scale and calculated as weight (kg)/height squared (m^2^).
- Dual-energy x-ray absorptiometry: assessment of total and regional fat mass, lean tissue mass, % body fat, bone mineral content, and bone density will be completed with a whole-body dual-energy x-ray absorptiometry scan [45].
- Waist and hip circumference: will be measured using standardized techniques [46].
- Resting blood pressure: will be measured after a 10-minute rest using standardized techniques [47].
- Food recalls: instruction on completing a 24-hour dietary recall will be provided to participants by a registered dietitian. Food records will be entered and analyzed for macronutrient and micronutrient composition by using the Automated Self-Administered 24-Hour Dietary Assessment program [28].
- Physical activity: Physical Activity Scale for the Elderly 11-item self-report of physical activity: leisure time and household activities and optional work or volunteer activities. Good reliability and validity; brief to reduce participant burden [30].
- A 6-minute walk distance: assessment of submaximal aerobic capacity, measured as the distance walked quickly during a period of 6 minutes [48].
- Gait speed: assessment of functional mobility, calculated from a 4-meter walk performed at self-selected and fast walking speeds, with the average of 3 trials used [49].
- A 30-second chair stand: assessment of functional lower extremity strength. Chair stand number will be recorded as the number of chair stands achieved in 30 seconds [50].
- Timed Get Up and Go Test: assessment of mobility, balance, walking ability, and fall risk. Measured by recording the time to get up from a fully seated position, walk around a cone placed 3 meters away, and return to a seated position, with the fastest of 2 trials used [49].
- Short physical performance battery: assessment of lower extremity function. A group of measures that combines the results of the gait speed, chair stand, and balance tests [51].
- Four-Square Step Test: test of dynamic balance and lateral mobility will be used to assess fall risk and dynamic balance [52]. Measured by the time to step over 4 canes set-up in a cross on the floor with the fastest of 2 trials used.
- Handgrip strength: Measure of isometric strength level of the hand and forearm. Hand grip strength of both arms will be assessed using a handheld dynamometer. Measures will be taken in triplicate to take the average of the 3 measures [53].

#### Peer-Led Diet and Exercise Intervention Procedures

Peer leaders at each site will serve as event organizers, offer guidance, and demonstrate healthy eating and exercise techniques at their respective sites. Participants will meet in groups biweekly for 12 weeks with the peer leaders to learn and discuss various content dealing with diet, exercise, and managing comorbidities (approximately 20-30 minutes per session) and to participate in a group exercise session (approximately 45-50 minutes per session). To ensure peer leaders implement the diet and exercise intervention as developed and the results reflect the true test of the program, peer leaders will complete weekly fidelity checks to document adherence to topics discussed, quality of delivery, major issues, component differentiation, and participant engagement [54]. Anticipated diet themes include budget-friendly nutrition for optimal aging: nutrition basics (ie, healthy dietary patterns and hydration), recipe modification, and mindful eating. Anticipated physical activity themes include American College of Sports Medicine (ACSM) physical activity recommendations (duration, intensity, and mode), as well as problem solving focused on how to safely exercise in their home environments. Other topics included are managing common comorbidities (ie, sleep behaviors, preventing and managing diabetes, avoiding excessive sitting, and managing stress) and available community resources. Sessions will be structured as a brief overview of the topic, followed by group discussion to allow the group to openly discuss the barriers to consuming a healthy diet and following the ACSM physical activity guidelines, as well as exchange ideas to improve their diet and exercise behaviors. This group dynamic provides the participating older adults an opportunity to build their social networks and provides supportive relationships to facilitate behavior change by meeting self-selected goals. These themes will not be prescriptive and will focus on topics for each week of the program.

#### Postintervention Assessments Measures and Subjective Surveys

All assessment battery measures described in Textbox 1 and web-based subjective assessments (surveys and questionnaires) described in Table 2 will be repeated after the 12-week intervention period. In addition, a postintervention exit interview (both quantitative and qualitative assessments) will be conducted by a research team member to obtain direct feedback on participant experience (ie, program enjoyment, lesson applicability, confidence and willingness to implement behavior changes, and identification of additional barriers not addressed).

### Outcomes and Analysis

The primary outcome will determine the feasibility and acceptability of the 12-week, peer-led intervention in older veterans with dysmobility (n=10 per site). Feasibility and acceptability of the peer-led intervention will be determined by assessing (1) reach (recruitment and retention), (2) adoption (satisfaction, perceived utility attendance, and engagement), (3) implementation (fidelity of intervention), and (4) estimated magnitude of potential impact on select outcomes (ie, energy balance, diet quality, and mobility). Secondary outcomes will assess (1) mobility using a standard battery of functional assessments (strength, balance, and endurance), (2) cardiometabolic risk factors (BMI, body composition, and resting blood pressure), and (3) psychological health (quality of life, fatigue, sleep quality, dietary intake and physical activity, and depression) before and after the 12-week intervention. Refer to Table 2 for web-based subjective assessments (surveys and questionnaires) and Textbox 1 for baseline and postintervention secondary outcome assessment measures.

The analysis plan includes feasibility (eg, recruitment, retention, and adherence) and acceptability using the RE-AIM (Reach, Effectiveness, Adoption, Implementation, Maintenance) framework [55]. A flowchart (ie, CONSORT [Consolidated Standards of Reporting Trials] chart) will be prepared to identify and summarize issues in recruitment and retention (record total numbers screened, numbers excluded with reasons for nonparticipation, timing and frequency of dropout, etc). Acceptable recruitment will be defined as 100% of the total targeted enrollment within 2 months of initiating recruitment efforts. Retention will be assessed by the frequency of dropouts; overall, 80% retention will be considered acceptable. An examination of attrition and exit interviews (quantitative and qualitative) will be conducted. The Usage Rating Profile-Intervention acceptability and feasibility subscales will be used to assess both peer leaders’ and participants’ responses to the intervention [56]. Successful adherence will be defined as participants completing at least 75% of all sessions. A random sampling of 30% of peer leader sessions will also be evaluated to determine whether peer leaders accurately covered all prescribed materials for each topic. The focus of a feasibility pilot study is to estimate the magnitude of the potential impact. Data will be inspected for out-of-range values, missing data, and internal consistency (when relevant). The clinical outcomes of interest are changes in diet quality and physical mobility after the 12-week intervention period. Similar changes in mobility with higher adherence and retention will indicate that long-term success is feasible for the program and warrants a trial with a longer duration. Summary statistics with CIs will be calculated to describe the average levels and trajectories of clinical outcomes over time. Baseline variables will be summarized, and frequency distributions will be examined for unusual data distributions or data points.

### Participant Safety and Minimizing Potential Risk

The risks of participating in the research program involve minor discomforts, but these are transient. The participants will undergo procedures that involve a mild to moderate degree of risk, including questionnaires, exercise training, weight loss, mobility assessment, and dual-energy x-ray absorptiometry scans. The interviews and questionnaires in this study are time-consuming but of minimal risk. Completion of physical activity is associated with the risk of cardiovascular complications such as chest pain, heart attack, or sudden death and complications related to stress and strain of muscles, twisted ankles, or falls. The American Heart Association consensus statement on exercise standards estimates that the acute risk of sudden cardiac arrest during exercise training in participants with known cardiac disease is approximately 1 event per 60,000 hours of aerobic exercise. The risk of exercise training is greater at higher exercise intensities. This risk can be offset by prescreening the participants with medical evaluations before exercise. In addition, all leaders will undergo a competency evaluation before leading any class. Competencies will include identifying signs and symptoms of a medical emergency, how and when to call VA emergency services in the gym setting, and who should be contacted for any adverse events that are not medical emergencies and for common injuries that occur during exercise. In addition, a trained research team member (exercise physiologist or other medical professional), certified by the American Heart Association Basic Life Support, will be present during all diet and exercise sessions. The research team member will step in to assist if the health or safety of participants or peer leaders is at risk. All instances of the research team members assisting peer leaders will be documented and assessed as part of the feasibility of the study. Minimal risks associated with weight loss and body weight will be monitored to ensure that the BMI does not fall below a healthy BMI of 18.5 kg/m^2^. Proper hydration guidelines will also be provided. In addition, there is a minimal risk of falling during the walking and mobility tests. A standby aid will always be present, and a gait belt will be used to increase safety when necessary. Ample rest periods will be provided to limit fatigue during testing. Finally, the 2 dual-energy x-ray absorptiometry scans will expose participants to a very small dose of ionizing radiation (0.6 mrem total) [45], which is well below the dose of a standard chest x-ray (8 mrem) [57]. Any dose of radiation could be potentially harmful; however, the radiation risk for the measurement of body composition is well within the established dosimetry of radiation guidelines and is not harmful to health or life [45].

### Data Management

All participants from stages 1 and 2 will be assigned a unique code stored in a password-protected database on a VA server that has a level and scope of security that equals or exceeds that established by the HIPAA Security Rules. Data collected during the study that is not captured electronically will be entered and stored in the VA Research Electronic Data Capture. The participants’ charts will be held in a locked room inside a locked cabinet at their respective facilities (Baltimore and Audie Murphy VAMCs). Access to individual participant numbers or personal identifiers will be limited to research team members who need these data to perform their roles in this study.

### Ethics Approval

Each site has been approved by the local Institutional Review Board (University of Maryland School of Medicine, HP-00097187; UT Health San Antonio, 21-890E) and VA Research and Development Committees in accordance with the ethical standards of the responsible committees on human experimentation and the Declaration of Helsinki. In addition, informed consent will be obtained from each participant, and they will be allowed to opt out. Phase 1 (Assessment of Barriers and Facilitators) is exempt from IRB approval under local IRB guidelines because of its web-based nature, minimal inclusion and exclusion criteria, and anonymous web-based surveys [58,59].

### Dissemination

The results of this study will be made available to health care professionals and the public through the National Library of Medicine PubMed Central website within 1 year after the date of publication. In addition, the findings of this study will be presented at scientific meetings. The study investigators will be responsible for writing all publications and will not use the services of professional writers.

## Results

The study was funded on January 1, 2022, with a projected data collection period of June 1, 2022, to December 31, 2023. Screening and enrollment of participants for stage 1 began on June 1, 2022.

## Discussion

### Overview

The purpose of this study is to develop and evaluate a novel, culturally tailored 12-week, peer-led diet and exercise intervention using a mixed methods approach that targets older veterans with dysmobility in 2 diverse urban areas with a high percentage of underrepresented minority veteran populations (Baltimore, Maryland, and San Antonio, Texas). Specifically, we anticipate that the results of this pilot study will identify barriers and facilitators of physical activity and dietary intake, develop a culturally sensitive peer-led diet and exercise intervention, and determine its feasibility in older veterans with dysmobility.

Previous peer-led diet and exercise interventions have been successfully implemented [18]; however, there are numerous limitations in their targeted population and design that make them suboptimal for older veterans with dysmobility. The multitude of interventions that have attempted to address diet and physical activity in older adults were not based on professionally led, evidence-based interventions with available long-term follow-up data. They have targeted only diet (mainly weight loss) or exercise (as opposed to both) and have emphasized walking but not following the United States Department of Agriculture dietary or ACSM exercise guidelines (ie, strength, endurance, and flexibility) for older adults [22]. Participants in these interventions are predominantly female [22], which may not translate to the veteran population, which is disproportionately older men [1]. Furthermore, few have focused directly on the unique needs of older veterans who often live in underserved areas without access to professional resources [60-62]. In addition, in many of these studies, minority populations are underrepresented. Because of the unique challenges faced by this population, including equitable access to high-quality foods (ie, food insecurity and food deserts) and high obesity rates [63], the ability to translate these interventions to other populations is challenging. Inclusion of minority populations who live in underresourced areas is critical to the development of a diet and exercise program because peer-led interventions targeting this population may require a different approach compared with predominately White or nonminority populations [64]. Furthermore, few studies have examined the determinants of dietary intake and physical activity among older veterans with limited physical functioning or dysmobility. Therefore, this study is designed to specifically assess the numerous limitations of previous studies. To our knowledge, this will be the first study to develop and implement a peer-led diet and exercise intervention that uses evidence-based interventions with long-term follow-up data that target both diet quality and physical activity in older veterans living in underserved areas with dysmobility, thus filling a significant knowledge gap in the current literature.

Physicians often have little time to offer support for lifestyle counseling, especially among older adults who often have multiple chronic conditions that require time-consuming medical management. Furthermore, diet and physical activity support from other trained professionals such as registered dietitians and exercise physiologists are often not readily available in low-income urban communities [60-62]. Peer-led interventions have been used in a variety of settings including support groups with people who share life experiences (eg, substance abuse and common illness), peer mentoring or coaching, and health promotion (eg, physical activity) [43] and have been shown to effectively help individuals achieve their desired goals [65]. Peer-based interventions have the potential to facilitate long-term health behavior changes, because peers often understand the obstacles that affect their communities [20]. In addition, peer leaders can serve as relatable role models and provide emotional and social support [26] to better connect and empathize with individuals of equal status and of similar age, background, and abilities [66]. Peer leaders offer a cost-effective opportunity to promote physical activity, healthy diet behaviors, and well-being in older adults by facilitating attention, retention, and motivation in recipients [67]. We have previously shown that using experienced, doctoral-level trainers to implement a diet modification intervention did not yield superior participant outcomes compared with training implemented by trained peer leaders [26]. Studies have also shown that peer-led interventions are as effective as professionally led interventions [67] and can lead to better program retention and adherence in older adults [68]. These data support the use of peer leaders to disseminate impactful education via lifestyle programs in a cost-effective manner. Therefore, the results of this study may identify a cost-effective and easily scalable strategy that increases long-term adherence to a program that improves the dietary quality and physical activity of older veterans and may reduce the risk of disability by maintaining mobility and preserving cardiovascular health with advancing age.

### Limitations

Despite its strengths, this study has potential limitations that should be considered. The behavior changes in diet and physical activity could possibly result from the veterans’ knowledge of participating in the intervention. Participants could change their behavior solely because of study participation, thus skewing the results positively. Selection bias is another limitation that could affect the results. Individuals willing to participate in the intervention would be aware of the health benefits of a healthy diet and exercise, and thus, they may be more prone to changing behaviors. This could result in the study findings not representing the true relationship between the intervention and outcomes within the average older veteran population with dysmobility. Recruitment of peer leaders may be difficult; therefore, we will recruit the current Gerofit and MOVE! participants who successfully made and maintained positive changes in their diet and exercise habits. The selected peer leaders may be concerned about the time commitment to learn the information and lead diet and exercise sessions. Developing the materials, resources, and training modules used in diet and exercise sessions will help mitigate the perceived time requirements. In addition, we will recruit 3 peer leaders per site to help distribute time commitments and prepare for possible peer leader attention. Another limitation is the response and sampling biases associated with self-reported subjective assessments (surveys and questionnaires). To minimize these biases, we will use a multimodal approach to clinical assessment measures and validated questionnaires that have demonstrated responses that measure what they claim to measure. Finally, the underlying design of a pilot study limits the interpretation of feasibility to only the participant’s characteristics and cannot be generalized beyond the inclusion and exclusion criteria.

### Future Directions

This pilot study will provide preliminary evidence for a larger grant application to examine the long-term effects and maintenance of health behaviors of a peer-led intervention trial on diet quality, energy balance, and mobility in a diverse sample of older veterans, a population more likely to self-report physical disability [14] and experience higher rates of obesity and comorbid conditions than the general population [14,15]. On the basis of the findings from this project, future clinical studies may address a broader array of clinical outcomes, as well as examine the potential impact on reductions in VA health care expenditure and use owing to improvements in health. Future trials are needed to validate these findings before broad-based acceptance and dissemination across VAs can occur. Thus, the proposed research will not only provide information to the veterans participating in this study but will also lay a foundation for future research targeting underrepresented minority veterans who lack access to specialty health care and are at increased risk of dysmobility and loss of independence.

### Conclusions

Our study design allows for a comprehensive assessment of complex personal and social phenomena. In doing so, the proposed study fills a critical knowledge gap in aging research by identifying and elaborating on barriers to a healthy lifestyle that may not have been captured previously in older veterans with dysmobility. Furthermore, peer-led interventions offer a potentially low-cost and easily scalable approach. This approach will encourage dietary and physical activity changes, with the goal of improving energy balance and ultimately increasing mobility in this at-risk population. As Baltimore, Maryland, and San Antonio, Texas, have a large number of typically understudied minority populations, the 2 sites will allow us to develop a program that specifically targets a group of older veterans typically excluded from lifestyle intervention trials (older urban men from minority populations with dysmobility).

## Appendix

Multimedia Appendix 1 Peer-review summary statement by Rehabilitation Research and Development SPiRE Program - Rehabilitation Research and Development Parent IRG - Office of Research & Development (RRDS) (National Institutes of Health, USA).

## Abbreviations

ACSM: American College of Sports Medicine
CONSORT: Consolidated Standards of Reporting Trials
HIPAA: Health Insurance Portability and Accountability Act
IRB: institutional review board
RE-AIM: Reach, Effectiveness, Adoption, Implementation, Maintenance
SPIRIT: Standard Protocol Items: Recommendations for Interventional Trials
VA: Veterans Affairs
VAMC: Veterans Administration Medical Center

## Data Availability

The final data sets underlying all publications resulting from the proposed research will be shared outside Department of Veterans Affairs. On request, a deidentified, anonymized data set will be created and shared pursuant to a data use agreement, appropriately limiting the use of the data set and prohibiting the recipient from identifying or reidentifying (or taking steps to identify or reidentify) any individual whose data are included in the data set.
