# Supplementary material for: Intervention in Older Urban-Dwelling Veterans With Dysmobility: Protocol for a Pilot Feasibility Clinical Trial
Source: JMIR Res Protoc. 2022 Jul 13;11(7):e39192. doi: 10.2196/39192 (PMC9330205; doi:10.2196/39192)
Supplement: Multimedia Appendix 1 [file resprot_v11i7e39192_app1.pdf]

SUMMARY STATEMENT

**PROGRAM CONTACT:**	**(Privileged Communication)**	***Release Date:***	**05/12/2021**
		Revised Date:	

	Application Number:	**1 I21 RX003739-01A1**
**Principal Investigator**	***Formerly:***	**1I21RX003739-01**

ADDISON, ODESSA

Applicant Organization: BALTIMORE VA MEDICAL CENTER

Review Group:	**RRDS**<br>**Rehabilitation Research and Development SPiRE Program**

Meeting Date:	**04/26/2021**	***RFA/PA:***	**RX21-003**
Council:	**AUG 2021**		
Requested Start:	**10/01/2021**		

Project Title:	**The Development and Implementation of a Peer-Led Diet and Exercise Intervention in Older Urban Dwelling Veterans with Dysmobility**
SRG Action:	**Impact Score:233**

**Human Subjects:**	**30-Human subjects involved - Certified, no SRG concerns**
**Animal Subjects:**	**10-No live vertebrate animals involved for competing appl.**
**Gender:**	**1A-Both genders, scientifically acceptable**
**Minority:**	**1A-Minorities and non-minorities, scientifically acceptable**
**Age:**	**1A-Children, Adults, Older Adults, scientifically acceptable**
	**Clinical Research - not NIH-defined Phase III Trial**

DESCRIPTION (provided by applicant):
Over the next ten years the share of Veterans age 65+ years will increase to over 50% of the total Veteran population. The ability to safely maintain mobility with aging is critical. Older Veterans with multiple chronic health conditions are more likely to experience mobility decline and our preliminary data show they also have decreased diet quality when compared to older non-Veterans. They further report that their reduced physical activity and poor dietary quality are related to a lack of access to exercise facilities, poor habits, and lack of motivation or boredom. While a multitude of interventions have attempted to address poor diet and physical inactivity in older adult; most have utilized resource-intensive professionally led diet OR exercise interventions, and few have focused on the unique needs of older Veterans. Peer support offers a potentially low-cost, easily scalable approach to encourage long-term dietary and physical activity change. In this proposal we seek to develop and pilot a 12-week peer-led lifestyle intervention that targets older Veterans with multiple chronic health conditions and dysmobility, in two diverse urban areas with a high percentage of underrepresented minority Veteran populations (Baltimore, MD and San Antonio, TX). We will accomplish this through two specific aims. Aim 1: Develop a theory-driven, peer-led nutrition and exercise intervention tailored for older Veterans with dysmobility. Aim 2: Determine the feasibility and acceptability of the peer-led intervention, to asses reach (recruitment, retention), adoption (satisfaction, perceived utility, attendance, engagement) and implementation (fidelity of intervention); as well as the estimated magnitude and potential impact on selected outcomes (i.e diet quality and mobility) in older 20 older Veterans with dysmobility and multiple chronic health conditions (N=10/site). Our results from Aim 2 will be compared to Gerofit data to determine long term potential of the project. With the successful development and pilot of this intervention we will be positioned to submit a multisite Rehabilitation and Research and Development (RR&D) Merit application focused on the use of peer-led interventions to improve long-term compliance to lifestyle intervention in older Veterans with multiple chronic conditions.

PUBLIC HEALTH RELEVANCE:
The majority of older Veterans do not meet the minimum healthy diet or physical activity recommendations, despite known benefits. Identifying novel ways to increase adherence to rehabilitation programs that improve dietary quality and physical activity may reduce the risk of disability in older Veterans. Peer-based interventions may be one method to facilitate lasting behavioral change since peers often share a common culture and knowledge about the problems that their community experiences. We propose to develop and evaluate a novel peer-led diet and exercise intervention that targets older Veterans with multiple chronic health conditions. Successful development and pilot of this intervention will provide the preliminary data for a larger multisite trial focused on the use of peer-led interventions to improve long-term compliance to lifestyle interventions in older Veterans.

CRITIQUE 1

Significance: This is a revised proposal for a two-site pilot study targeting high risk Veterans with dysmobility and multiple chronic health conditions (MCC). The revised proposal is mostly very responsive to the critiques from the previous review. The project has high significance, as it offers the potential to find interventions that would have a long-term adherence by Veterans at high risk for dysmobility and loss of independence due to MCC. Also, if the peer-led approach is successful, it has important implications for application to other challenging and important behavioral change issues as well.

Approach:
Strengths:
- This is a well-planned study with clearly delineated methods and a strong analytical plan.

- The investigators have provided changes in the approach that meet the recommendations from the first review and strengthen the proposal.
- These include the addition of a detailed and more comprehensive approach to selecting and training the peer leaders, including a much more complete plan for assuring participant safety.
- Other improvements include the addition of appropriate mental health exclusions and allowances for assessment of opinions about the impact of gender/role differences for future trials.

Weaknesses/Questions: One important concern remains regarding the requirement that Veterans complete a 5-day diet record, inputting their own data using an Automated Self-Administered 24-hour Dietary Assessment Tool (the ASA24). Given the likelihood that some will struggle more than others, it is strongly advised that the investigators look for ways to individually assist every participant so as to get the most accurate food intake report, since diet quality is one of your most important outcomes. This will also help to assure that a “learning effect” doesn’t skew the diet intake reports between the first and second time point.

Innovation: Innovation hinges on the study of the unique needs of high-risk Veterans with dysmobility and MCC, as well as those under-represented and of lower education level.

Investigator Qualifications: The Principal Investigator (PI) (New and Early Stage) is well positioned and strongly qualified for taking on this project. There are also a number of supportive, experienced co-investigators at both sites. I did not see a response to the comment in the first review (R3) that “there should be a team member with expertise in instruction design and online education”.

Environment: No concerns. Environments are highly supportive.

Design/Scope: The proposed design is appropriate for the SPiRE mechanism and should be attainable in the time available. The recruitment plan is reasonable and the feasibility analysis leading from Aim 1 into Aim 2 is well conceptualized.

Human Subjects: Applicable and appropriate.

Inclusion of Women, Minorities and Children: Applicable and appropriate.

Critique of Vertebrate Animals Section:

	Yes	No
Research with vertebrate animals?		X

Biohazards and Radioisotopes: Not applicable.

Budget: No concerns.

Data Management and Access Plan (for data sharing, unscored)**:** Applicable and appropriate.

Overall Strengths:
- Highly responsive to comments from the first review, as detailed above, and also below under “Weaknesses”.
- The project has high significance, with the promising potential of the peer-led intervention.
- The approach is well delineated, with a strong analysis plan.

- Strong investigative group and environments.

Overall Weaknesses: It is true that the nature of the SPiRE mechanism does not permit confirmation that the peer-led approach will achieve long-term adherence to lifestyle interventions in the target population. (This was a major concern from the first review.) However, the investigators have done an excellent job of maximizing their potential to do so by planning to compare their outcomes to long-term adherence patterns in Gerofit and MOVE!. They will also conduct detailed qualitative interviews with those who drop out before the study ends to determine the causes for attrition.

CRITIQUE 2

Significance: This is a resubmission. Investigators seek to develop and pilot a 12-week peer-led lifestyle intervention that targets older Veterans with multiple chronic health conditions and dysmobility in two diverse urban areas with a high percentage of underrepresented minority Veteran populations (Baltimore, MD, and San Antonio, TX). Aim 1 involves developing the intervention and Aim 2 involves determining the feasibility and acceptability in a 20-person pilot.

It is not clear how some of the reasons for lower physical activity and poorer diet among people with dysmobility (e.g., lack of access to exercise facilities, disabling conditions and medications) will be ameliorated via a peer support program.

Approach: Aim 1a will involve an online survey. Our experience in older adults with disabilities is that many do not have computer skills and/or internet access. The investigators may want to consider other modes to administer their survey (e.g., paper or phone).

In the Introduction, the investigators state that they will interview individuals who drop out to understand their reason for dropping out. While I think that the investigators should try to interview those who drop out, in my experience, those who drop out of an intervention often refuse further participation in any form, including exit interviews.

Not clear how Aim 1a (survey and interviews) will inform Aim 1b.

The peer leader training is a bit confusing. The investigators state that the peer leaders will be selected because they demonstrate an understanding of the importance of diet and exercise. As part of training “they will learn the importance of physical activity and healthy eating.” They will also learn about social determinants of health and discuss solutions. I would like to better understand what the evidence-based solutions are. What will the solutions be for someone who lives in a neighborhood where they do not feel it is safe to walk and they do not have transportation?

It is unclear if there is a single peer for each group and if they are the sole leader. Do they only meet with attendees in the groups? Is there a plan for communication outside the groups?

There is insufficient information about the qualitative data analysis plan. Also, it was not clear how the qualitative assessment directly informs the pilot intervention. It would be helpful if the investigators gave specific examples of how they would use the information to inform the design of the pilot study.

Aim 2 – the investigators did not mention an inclusion criterion related to not having a healthy diet or wishing to change their diet and increase their physical activity, but it seems as if those not meeting guidelines/underactive would be their target population.

The investigators mention a screening visit, but it is not clear what inclusion/exclusion criteria are assessed via the physical examination.

Aim 2 assessment seem focused on mobility rather than physical activity, yet the background discusses the different components of fitness. It would have been helpful if they tied these together more directly. There appears to be no objective or self-reported measure of physical activity. This is a limitation.

Table 3 notes that there will be 5 people per site while the text states that there will be 10 people per site.

The proposal states, "The focus in a feasibility pilot study like this is to estimate the magnitude of potential impact." Well-respected researchers have presented strong arguments that while pilot studies are useful for feasibility and acceptability, because of their small size, they are not good for estimating magnitude of benefit. That is the job of the full-scale trial. See: Andrew C Leon [1], Lori L Davis, Helena C Kraemer. The role and interpretation of pilot studies in clinical research. J Psychiatr Res. 2011 May;45(5):626-9.

Not clear how some of the exclusion criteria (e.g., high cardiovascular risk (poorly controlled hypertension >160/100, class IV chronic heart failure, symptomatic angina at rest, or syncope in the last year without known resolution of cause); use of home oxygen; and contraindications to an exercise intervention will be assessed. The human subjects document specifically assesses administration of the mini-mental assessment, but not any others.

The targeted/planned enrollment tables and recruitment tables only list 20 people. It is not clear why those included in Aim 1 activities are not included.

Innovation: This research fills an important gap, as it targets both physical activity and diet and includes a population typically excluded from research (those with disabilities or multiple chronic health conditions).

Investigator Qualifications: The investigator is a current CDA-2 awardee, which will be completed in July 2021. She has a strong background in mechanistic studies but does not have experience with peer support interventions or qualitative data collection. Co-I's have experience in biostatistics and epidemiology (Sorkin), exercise in older adults (Katzel), exercise/diet (Parker, Serra), implementation (Kilpela), and frailty (Espinoza). It is not apparent that anyone on the team has previously created and led a peer-led intervention.

Environment: Excellent. No concerns.

Design/Scope: See above.

Human Subjects: No concerns.

Inclusion of Women, Minorities and Children: This was mentioned, but there does not appear to be a specific plan to ensure recruitment of women and minorities. The investigators do note that Baltimore (one of the sites) has a high proportion of individuals who are Black and San Antonio, while it is predominantly Caucasian, a large proportion are Hispanic. It is not clear that these statistics are representative of the population targeted (Veterans using VA).

Critique of Vertebrate Animals Section:

	Yes	No
Research with vertebrate animals?		x

Biohazards and Radioisotopes: No comment.

Budget (unscored)**:** Slight concern about low FTE for the PI and all Co-Is.

Data Management and Access Plan (for data sharing, unscored)**:** No comment.

Overall Strengths: Identifying ways to increase physical activity and improve diet quality in people with dysmotility is important.

Overall Weaknesses:
- It is not clear how some of the reasons for lower physical activity and poorer diet among people with dysmobility (e.g., lack of access to exercise facilities, disabling conditions and medications) will be ameliorated via a peer led program.
- Unclear screening plan; including how exclusion criteria will be assessed.
- The plan to use the pilot data to estimate the magnitude of potential impact is problematic and could important problems if the pilot data are used for power/sample size calculations for a future trial. See the reference included above.
- The conceptual model for the peer leaders lacks clarity. One justification for having peers as leaders is to decrease costs and increase scalability, but there is a lack of clarity about whether the leaders are peers, and the model is one of peer support, or simply lay leaders without a support component.
- Plan for analysis of the qualitative data is underdeveloped.

CRITIQUE 3.

Significance: The peer-led nutrition and exercise intervention is a very important area. The proposed study is supported by multiple pilot studies to support sustainability of the intervention.

Approach: Well formulated hypothesis and research questions. The aims are answerable based on the purposed methods. The two phases of the study are well designed.

Innovation: The study has substantial benefit for the Veteran's health care. The study has potential to develop into a full-scale research project.

Investigator Qualifications: Research team is qualified to conduct the proposed study.

Environment: All necessary resources are present and available. Great institutional supports.

Human Subjects: The proposal addressed all five-key consideration for the human subjects.

Inclusion of Women, Minorities and Children: Applicable and Appropriate.

Critique of Vertebrate Animals Section:

	Yes	No
Research with vertebrate animals?		X

Biohazards and Radioisotopes: Not applicable.

Budget: The project timeline, costs allocation, and personnel costs are appropriate.

Data Management and Access Plan (for data sharing, unscored)**:** The plan for sharing research data is consistent with other aspects of the application.

Overall Strengths:
- The proposed study is supported by multiple pilot studies.
- The pilot studies showed promising sustainability of the program.
- The 2 phases study is well designed.

Overall Weaknesses: No major weaknesses.

SCIENTIFIC REVIEW OFFICER

BENTON-GROVER, KRISTY
MANAGEMENT AND PROGRAM ANALYST
DEPARTMENT OF VETERANS AFFAIRS
VETERANS HEALTH ADMINISTRATION
OFFICE OF RESEARCH AND DEVELOPMENT
REHABILITATION RESEARCH & DEVELOPMENT SERVICE
WASHINGTON, DC 20420
